# One Face, Three Solutions: Structural Convergence in PD-L1 Inhibition across Antibodies, Macrocycles, and Small Molecules

**DOI:** 10.1021/acs.jmedchem.6c00674

**Published:** 2026-07-02

**Authors:** Imma Capriello, Thiago Moreira Pereira, Gustavo Barbosa Reis, Vishwanatha Thimmalapura Marulappa, Katarzyna Magiera-Mularz, Jacek Plewka, Tad A. Holak, Alexander Dömling

**Affiliations:** † Czech Advanced Technology and Research Institute (CATRIN), Palacký University Olomouc, Slechtitelů 27, 77900 Olomouc, Czech Republic; ‡ Institute of Molecular and Translational Medicine, Faculty of Medicine and Dentistry, Palacký University and University Hospital Olomouc, Hněvotínská 1333/5, 77900 Olomouc, Czech Republic; § Department of Chemistry—Institute of Environmental, Chemical and Pharmaceutical Sciences, Federal University of São Paulo, Prof. Artur Riedel, 275—Jardim Eldorado, 09972-270 Diadema, São Paulo, Brazil; ∥ Faculty of Chemistry, 37799 Jagiellonian University, Gronostajowa 2, 30-387 Krakow, Poland; ⊥ Department of Biomedical Chemistry, Faculty of Chemistry, University of Gdańsk, 80-308 Gdańsk, Poland

**Keywords:** PD-L1, PD-1, protein protein interaction, drug modality, hot spot, mAb, cyclic peptide, small molecule

## Abstract

Protein–protein interactions dominated by large, flat interfaces are widely considered challenging drug targets. The programmed cell death protein-1/programmed death ligand-1 (PD-1/PD-L1) immune checkpoint exemplifies this problem, as the interaction is mediated by an extended β-sheet surface lacking deep pockets. Despite this, PD-L1 has been successfully inhibited by chemically distinct modalities, including antibodies, macrocyclic peptides, and small molecules. Here, we present a comparative, structure-driven analysis of PD-L1 complexes deposited in the Protein Data Bank and demonstrate a striking convergence: all effective inhibitors engage the same CC′FG β-sheet face of PD-L1. Antibodies directly occlude this surface, macrocyclic peptides such as pAC65 reproduce antibody-like surface coverage in a compact and preorganized scaffold, and biphenyl small molecules neutralize the same epitope indirectly by inducing PD-L1 homodimerization. This unified structural framework reveals modality-agnostic design principles for targeting flat immune checkpoint PPIs. This Perspective provides a unified structural framework for understanding PD-L1 inhibition across clinically tested antibodies, macrocyclic peptides, and small molecules. Both visualizing and quantitatively comparing interface overlap, hotspot conservation, and buried surface area, the work demonstrates that distinct inhibitory modalities converge on the same functional CC′FG hotspot region while employing fundamentally different neutralization mechanisms. These findings establish structure-guided principles for the rational design of next-generation PD-L1 modulators across diverse therapeutic modalities.

## Graphical abstract

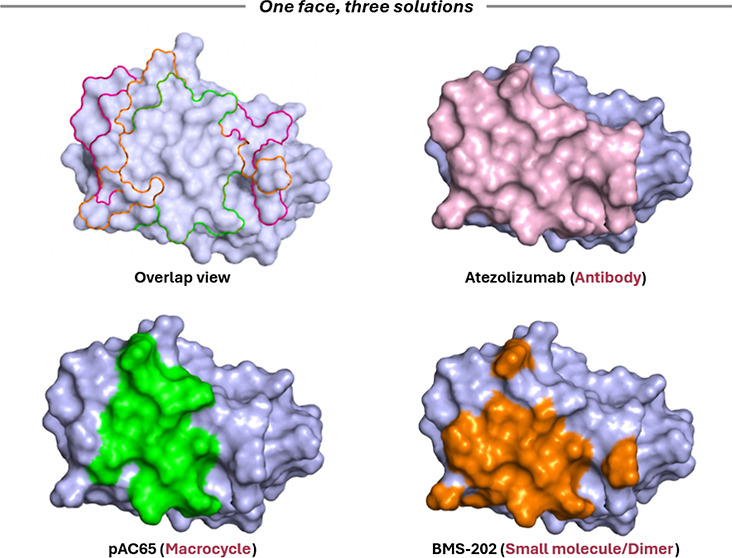

## 1 Introduction

Immune checkpoint blockade has triggered a fundamental shift in cancer therapy, representing the most consequential advance in the field in recent decades and establishing the PD-1/PD-L1 axis as a central clinical target (Figure 1).^1,2^ Although clinically effective, PD-1/PD-L1 antibodies are associated with immune-related adverse events that vary by target and agent, poor tumor penetration, high costs, and intravenous administration underscoring the ongoing need for modalities with potentially different biodistribution and toxicity profiles. From a molecular perspective, this success is remarkable. PD-1 and PD-L1 interact through a face-to-face contact of two IgV domains, forming a broad, relatively flat β-sheet-dominated interface.^3^ Classical medicinal chemistry would predict such a surface to be poorly druggable.^4–6^ Nonetheless, PD-L1 has emerged as a rare example of a flat protein–protein interaction that can be modulated by multiple, chemically distinct modalities.^7^ These approaches are often discussed in isolation, emphasizing differences in size, chemistry, or mechanism.^8^ Here, we argue that this view obscures a deeper structural unity. By aligning PD-L1 complexes with PD-1, antibodies, macrocyclic peptides, and small-molecule dimerizers, a single epitope—the CC′FG β-sheet face of PD-L1—emerges as the universal focal point of inhibition. The differences between modalities lie not in where they bind, but in how they neutralize this surface.

**Figure 1:**
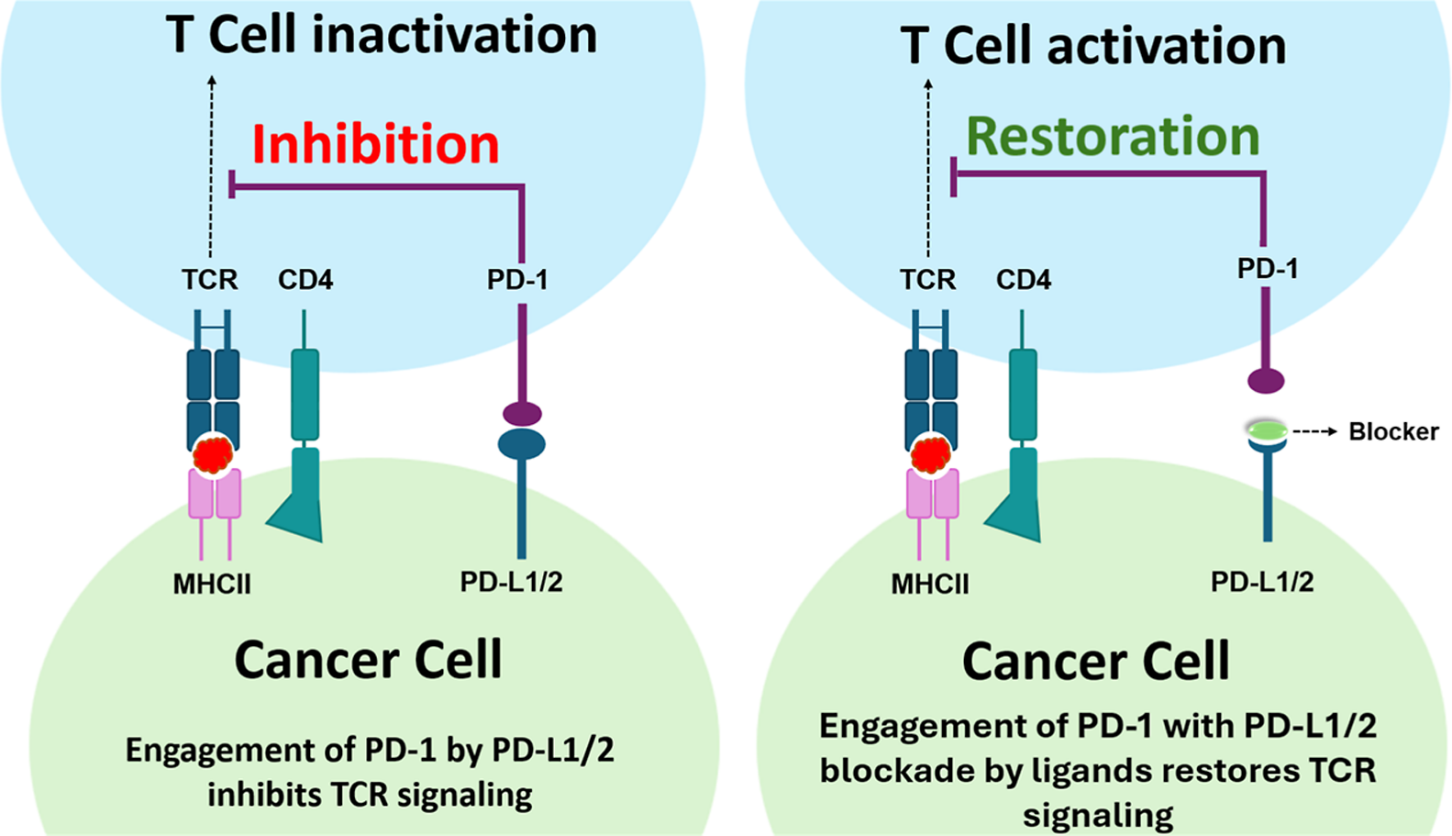
Inhibition of PD-1/PD-L1 interaction leads to a new immune checkpoint in cancer biology. Engagement of PD-1 on T cells by PD-L1 expressed on tumor cells or antigen-presenting cells delivers an inhibitory signal that suppresses T-cell activation, proliferation, and cytokine production, thereby promoting tumor immune evasion. Therapeutic blockade of the PD-1/PD-L1 interaction restores antitumor immunity by preventing inhibitory signaling at the immunological synapse. This pathway constitutes the central molecular target of immune checkpoint inhibition strategies discussed in this Perspective.

## 2 PD-1/PD-L1 as a Prototypical Flat Protein–Protein Interaction

The PD-1/PD-L1 crystal structure defines the structural constraints that govern all subsequent inhibitor design.^3^ PD-1 engages PD-L1 through the CC′FG β-sheet face, forming an extended interface characterized by distributed hydrophobic contacts and peripheral polar interactions (Figure 2). Throughout this Perspective, the term “CC′FG face” refers to the flat β-sheet interaction surface formed primarily by the CC′, F, and G strands of the PD-L1 IgV domain. No dominant pocket is present, and binding energy is spread over a large surface area. Two features are particularly consequential. First, partial engagement of the interface is insufficient to compete with PD-1; effective inhibition requires near-complete surface neutralization. This is because the PD-1/PD-L1 interaction is a distributed interface, not a hot-spot-driven one, such as p53/Mdm2. Second, the PD-L1 IgV domain presents a rigid surface with high shape complementarity exhibits limited conformational plasticity upon PD-1 binding, restricting opportunities for induced-fit pocket formation. The geometry of the PD-1/PD-L1 interface leaves little scope for allosteric modulation, and no functionally validated distal allosteric site on PD-L1 capable of disrupting PD-1/PD-L1 binding has been convincingly demonstrated to date. As a result, the PD-1/PD-L1 complex provides an unambiguous structural reference frame: any inhibitor capable of disrupting signaling must intersect this same CC′FG face (Figure 2).^3^

**Figure 2:**
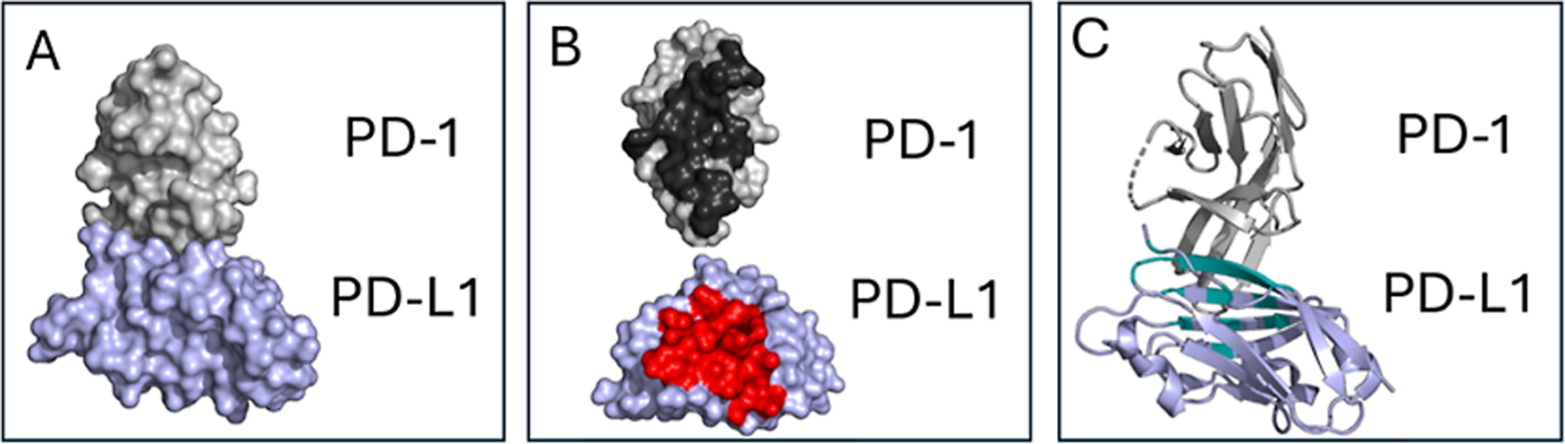
β-Sheet face is the interaction hotspot of the PD-1/PD-L1 complex and the corresponding residues on PD-L1 interface (PDB 4ZQK). (A) Crystal structure of human PD-1 bound to human PD-L1. PD-L1 is shown as a blue surface and PD-1 as a gray surface. The complex is formed through an extended, relatively flat IgV–IgV interface dominated by β-sheet complementarity, defined by the CC′FG β-sheet face. (B) Opening of the complex exposes the CC′FG faces of PD-L1 (red) and PD-1 (black), which together constitute the complete biologically relevant epitope for immune checkpoint signaling and define the structural reference frame used for all subsequent overlays. The total buried solvent-accessible surface area (BSA), calculated as BSA = (SA_A + SA_B – SA_complex)/2 using isolated binding partners, is approximately 1536 Å^2^. (C) Cartoon representation of the PD-1/PD-L1 complex, highlighting in green the flat and extended IgV–IgV interaction geometry.

## 3 Antibodies Targeting PD-L1: Structural Redundancy as Validation

Currently 12 antibodies acting against PD1 or PDL1 are in clinical use. Therapeutic antibodies against PD-L1 provide the first confirmation of this constraint. Structures of PD-L1 bound to antibodies such as atezolizumab and durvalumab reveal an almost invariant epitope (Figure 3).^9–12^ Despite differences in antibody sequence and CDR composition, all antibodies engage the CC′FG β-sheet face used by PD-1. At the atomic level, antibody paratopes deploy aromatic residues to blanket the hydrophobic core of the PD-L1 surface, frequently stacking against Tyr56 and Tyr123, while polar and charged residues engage the rim of the interface, including the Arg113/Arg125 region. These structures establish a “gold-standard” geometry for PD-L1 inhibition: extensive surface coverage, aromatic-rich contacts, and peripheral electrostatic locking.

**Figure 3:**
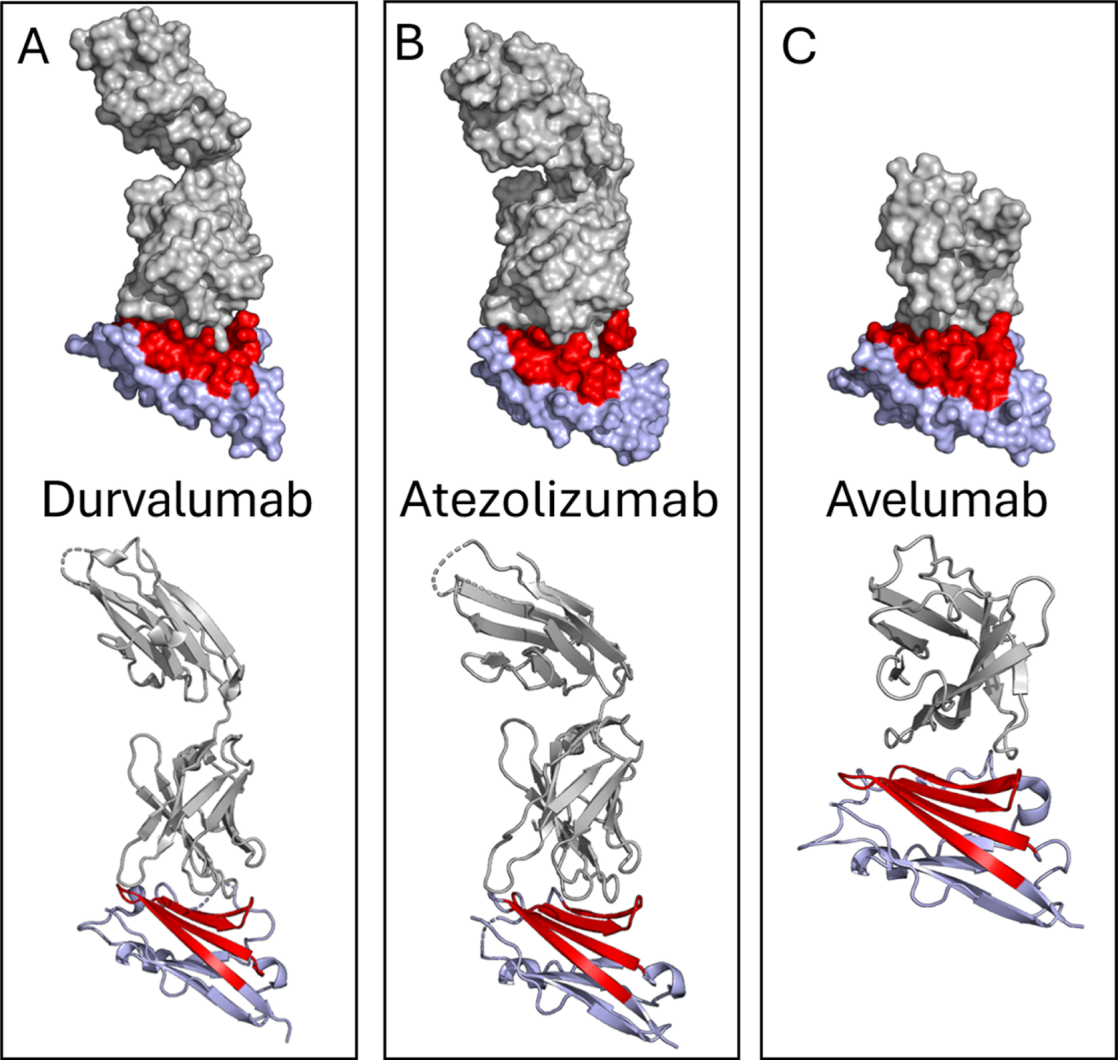
Different therapeutic antibodies binding on the same PD-L1 CC′FG face. (A) Duravalumab-PD-L1 (PDB: 5X8L); (B) Atezolizumab–PD-L1 (PDB: 5XXY); (C) Avelumab–PD-L1 (PDB: 5GRJ). Structural comparison of PD-L1–antibody complexes highlighting epitope convergence across clinically validated antibodies. Top row: PD-L1 is shown in a fixed orientation as a light blue surface, with the CC′FG β-sheet face buried upon antibody binding indicated in red. Antibody Fabs are rendered as gray surfaces to emphasize direct surface occlusion of the PD-1 interaction site. Bottom row: the same complexes are shown in cartoon representation, illustrating the relative orientation and approach of the antibody Fabs (gray) toward the PD-L1 IgV domain (light blue), with the CC′FG β-sheet highlighted in red. The total buried solvent-accessible surface areas are approximately 2114 Å^2^. Despite differences in antibody sequence and paratope architecture, all antibodies engage the same CC′FG β-sheet face used by PD-1, demonstrating epitope invariance across therapeutic PD-L1 antibodies.

## 4 Macrocyclic Peptides: The BMS-986189 Macrocycle (pAC65) as a Minimal Antibody Paratope

BMS-986189 is a clinically advanced macrocyclic PD-L1 inhibitor currently in Phase 2 clinical evaluation.^13,14^ The optimized macrocyclic peptide exhibited sub-nM PD-L1 affinity, in vivo efficacy together with substantially improved serum stability, prolonged half-life, and oral bioavailability. The cocrystal structure of its macrocyclic binding core, pAC65, bound to PD-L1 provides a unique opportunity to compare a clinically relevant peptide modality directly with antibodies and small-molecule inhibitors (Figure 4).^15^ pAC65 offers a striking demonstration that antibody-like geometry can be achieved in a much smaller scaffold. Structural alignment of the pAC65–PD-L1 complex with antibody–PD-L1 structures shows that pAC65 binds parallel to the CC′FG β-sheet face (Figure 4), occupying the same plane as PD-1 (Figure 2) and antibodies (Figure 3). Other cocrystallized cyclic peptides show similar structural features.^16^

**Figure 4:**
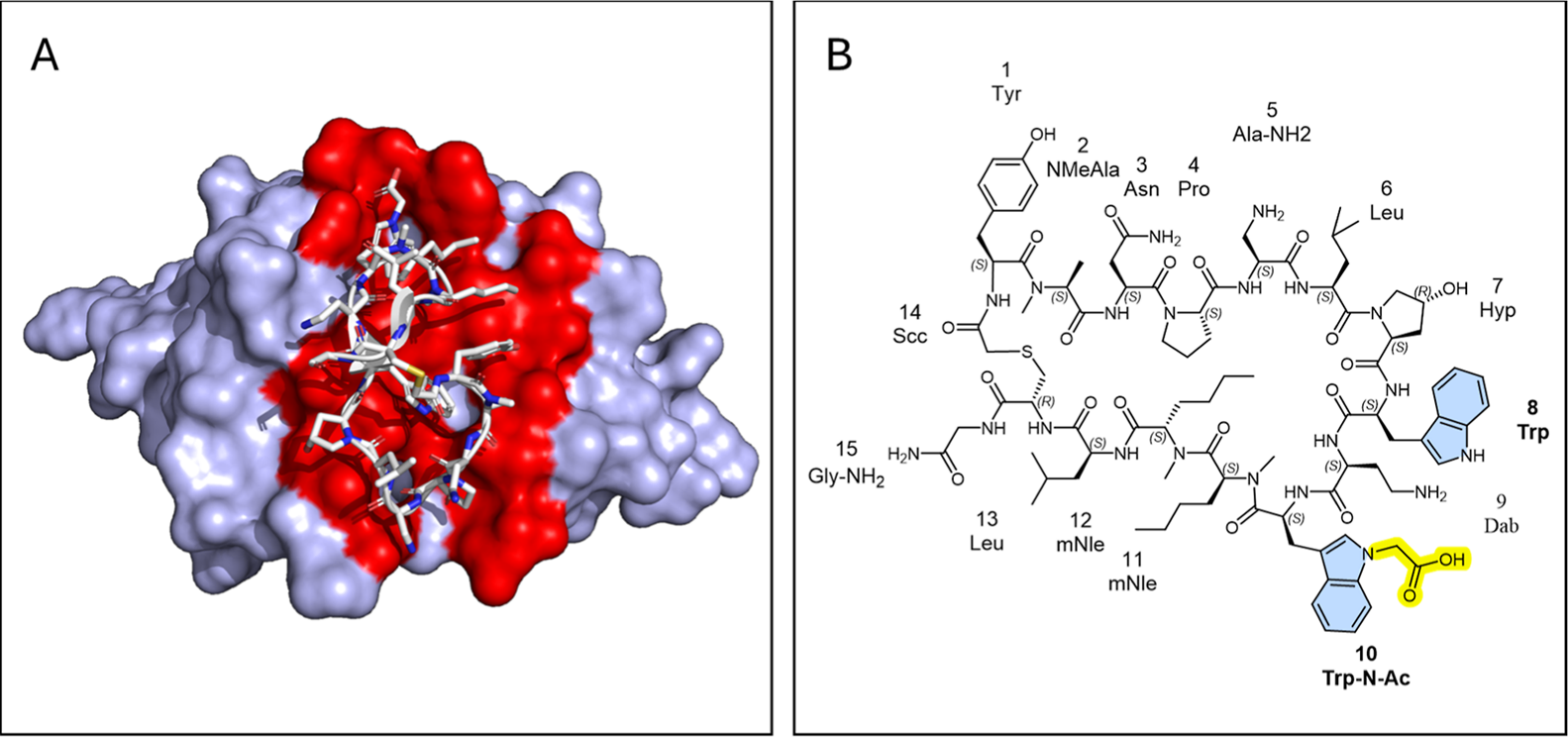
Macrocyclic peptide pAC65, the binding core of BMS-986189, reproduces antibody-like binding geometry (PDB: 8ALX). (A) PD-L1 (surface presentation) bound to the macrocyclic peptide pAC65 (stick representation), the buried surface area shown in red. pAC65 binds parallel to the PD-L1 surface and occupies the same binding plane as PD-1 and antibody paratopes, demonstrating convergence on an antibody-like mode of surface engagement despite its reduced size. The total buried solvent-accessible surface area is approximately 663 Å^2^. (B) 2D structure of the macrocyclic peptide pAC65, with the key tryptophan residues (blue) and the acyl side chain (yellow) highlighted, which together act as aromatic anchors for surface recognition and binding to PD-L1. The peptide adopts a preorganized conformation stabilized by a network of intramolecular hydrogen bonds, which enforces the geometry required for efficient coverage of the CC′FG face and recapitulates essential features of antibody paratope architecture within a compact synthetic scaffold.

Despite its reduced size, the BMS-986189 macrocycle pAC65 reproduces the essential interaction logic of an antibody paratope. Two indole-based residues act as dominant hydrophobic anchors, engaging the same PD-L1 hot spots contacted by antibody CDRs, while an anionic acetyl substituent interacts with the positively charged rim of the interface. Conformational preorganization, enforced by macrocyclization and backbone modification, minimizes entropic penalties and effectively “freezes” the peptide in a binding-competent conformation. Structurally, pAC65 can therefore be viewed as a single, optimized antibody CDR loop transplanted into a synthetic macrocyclic framework. In addition to macrocyclic peptides, several linear PD-L1-targeting peptides and peptide-derived binders have also been reported.^17,18^ Although generally characterized by lower conformational preorganization and reduced proteolytic stability compared with macrocycles, these ligands nevertheless reinforce the central structural observation of this Perspective: productive PD-L1 recognition consistently converges on the CC′FG β-sheet face. Available structural and computational analyses suggest that even linear peptide binders preferentially exploit the same hydrophobic hot spots and polar rim interactions that dominate antibody, macrocyclic peptide, and small-molecule recognition. Thus, despite substantial architectural differences, the underlying geometric logic of PD-L1 neutralization appears conserved across peptide classes.

## 5 Small-Molecule PD-L1 Dimerizers: Neutralization by Burial

Small-molecule biphenyl-based inhibitors of PD-L1 adopt a distinct but structurally revealing strategy.^19,20^ These classes of inhibitors lead to a dimerization of the extracellular domain of PD-L1.^21^ These compounds do not directly occlude the PD-1 binding surface; instead, they induce PD-L1 homodimerization, burying the CC′FG face within a newly formed protein–protein interface (Figure 5). In these complexes, the small molecule occupies a shallow hydrophobic groove that emerges only upon dimer formation.^22^ Stabilization of this dimer removes the functional epitope from solution and prevents PD-1 engagement. Although mechanistically indirect, this approach still relies on aromatic surface coverage analogous to that used by antibodies and peptides. The requirement for extensive hydrophobic interactions rationalizes the narrow structure–activity relationships and developability challenges associated with PD-L1 dimerizers.

**Figure 5:**
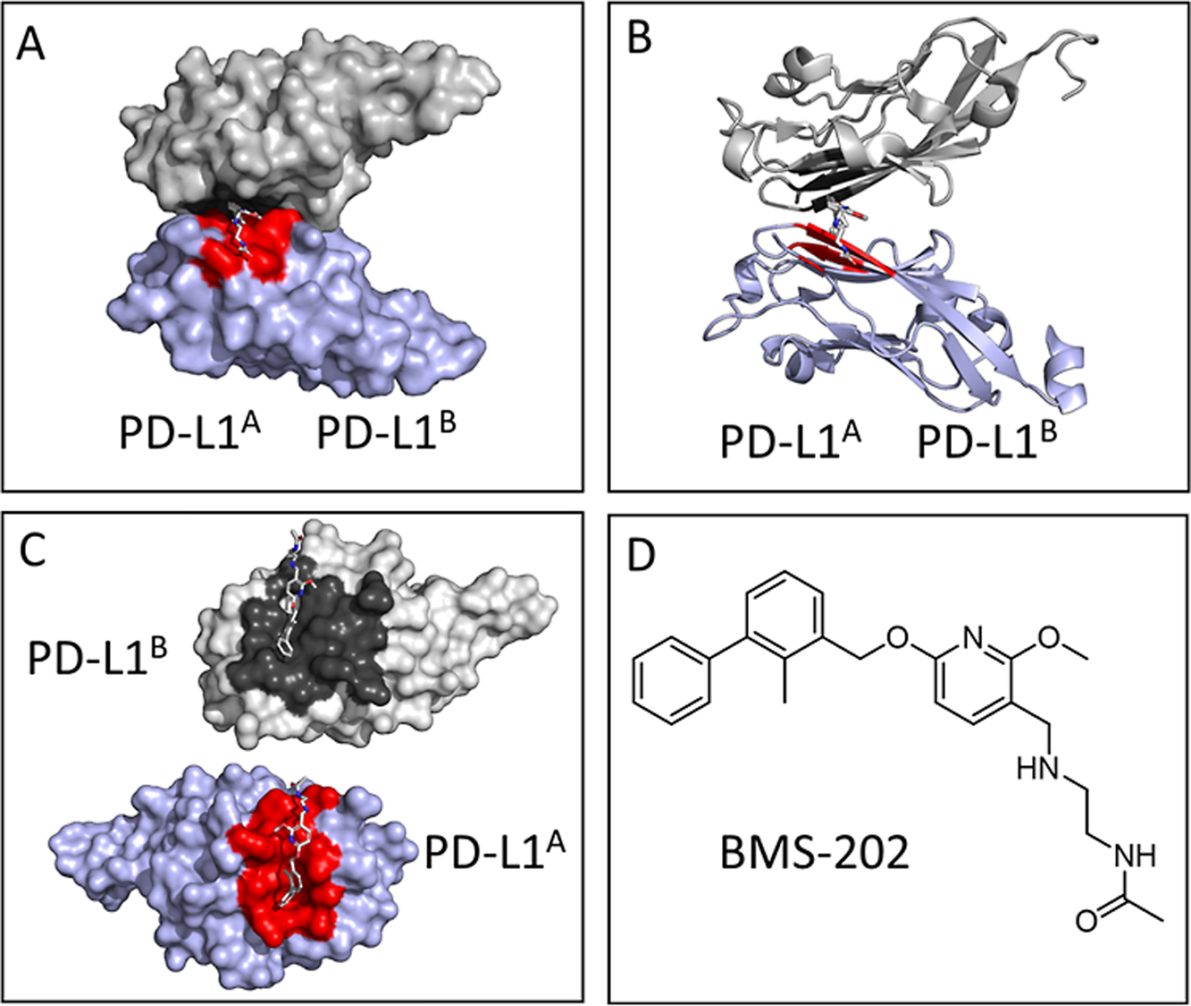
BMS-202 as a small-molecule–induced PD-L1 dimerization buries the functional epitope. (PDBs: 5J89). (A) Surface representation of the PD-L1 homodimer induced by the biphenyl small-molecule inhibitor. The two PD-L1 protomers are shown in light blue (monomer A) and gray (monomer B). The CC′FG β-sheet surface buried upon dimerization is highlighted in red for monomer A and in black for monomer B, illustrating the complementary burial of the functional epitope on both protomers. The small-molecule dimerizer is shown as gray sticks at the dimer interface. The total buried solvent-accessible surface area is approximately 963 Å^2^. (B) Cartoon representation of the PD-L1 homodimer, emphasizing the relative orientation of the two IgV domains and the burial of the CC′FG β-sheet faces (highlighted in red) within the dimer interface. (C) Opened view of the same complex, highlighting the extent of surface burial on each PD-L1 protomer upon dimer formation. The small molecule occupies a hydrophobic groove formed at the protein–protein interface, stabilizing the PD-L1–PD-L1 assembly and sequestering the CC′FG face from solvent exposure. (D) 2D structure of the biphenyl inhibitor BMS-202. Together, these structures illustrate an indirect mechanism of PD-L1 neutralization, in which small-molecule–induced dimerization buries the PD-1 interaction surface rather than directly occluding it, in contrast to antibody- and peptide-based inhibitors.

## 6 Structural Convergence across Modalities

When antibody, macrocyclic peptide, and small-molecule complexes are superimposed on PD-L1, the apparent diversity of PD-L1 inhibitors collapses into a small number of recurring structural solutions (Figure 6). Regardless of modality, all effective inhibitors converge on the same region of PD-L1 - the CC′FG β-sheet plane on the IgV “front face” - and none engage distal allosteric sites. Thus, despite their chemical and architectural differences, antibodies, BMS-986189-like macrocycles, and small molecules all exploit a common geometric constraint imposed by the PD-1/PD-L1 interface. Across these modalities, a shared interaction logic emerges, defined by three conserved elements. First, all potent inhibitors deploy two hydrophobic or aromatic anchors positioned to occupy the two shallow clefts on the CC′FG face. In pAC65, these anchors are Trp8 and the acylated TrpNAc10, which stack against Tyr56 and Tyr123 of PD-L1. Antibodies achieve an analogous interaction through aromatic residues in their CDR loops, which dominate the central packing against the same surface hot spots. Small-molecule biphenyl inhibitors similarly place two coplanar aromatic rings across this face, engaging Tyr56 and Tyr123 and thereby recapitulating the aromatic surface coverage provided by peptide and antibody binders. Second, effective inhibitors incorporate an anionic or polar “directionality handle” that engages the conserved Arg patch on PD-L1 (notably Arg113 and Arg125). In pAC65, the acetic acid moiety of TrpNAc10 forms salt bridges with this Arg cluster. Antibodies repeatedly exploit the same region through polar and charged CDR residues; for example, atezolizumab engages Arg113/Arg125 in conjunction with additional rim contacts such as Glu58. Biphenyl inhibitors, although chemically simpler, also position adjacent substituents to form salt bridges or polar interactions with this Arg patch. Third, all modalities employ a peripheral polar interaction network to enhance affinity and specificity without compromising the core hydrophobic packing. In pAC65, this is achieved through strategically placed Dab and Asn residues that form direct and water-mediated hydrogen bonds, aided by backbone turns that preorganize the binding surface. Antibodies implement the same principle through diversified polar residues in their CDRs, which decorate the periphery of the binding interface. Despite this shared interaction logic, the modalities differ in how they functionally disable PD-L1. Antibodies occlude the interface through extensive surface coverage, directly preventing PD-1 engagement. Macrocyclic peptides also block the interface but, in addition, impose conformational rigidity that effectively “freezes” PD-L1 in a nonproductive state. Small-molecule biphenyl inhibitors, by contrast, bury the interface indirectly by stabilizing a PD-L1 homodimer. These distinct mechanisms—block, freeze, or bury—represent alternative responses to the same underlying geometric constraint of the CC′FG β-sheet face.

**Figure 6:**
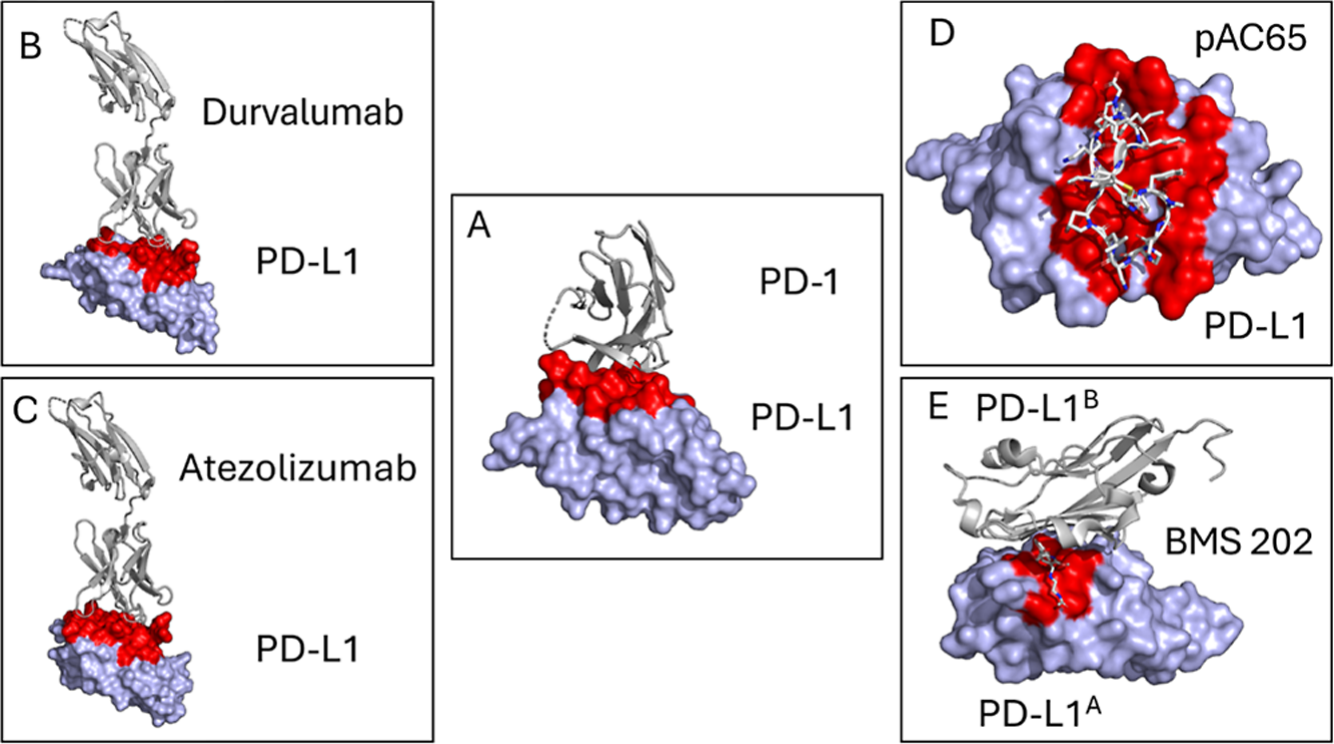
Overview of structural convergence of PD-L1 inhibition across molecular modalities which all inhibitors interact in the same β-sheet face (PDBs: 4ZQK, 5XXY, 8ALX, 5J89). (A) Reference view of the PD-1/PD-L1 complex (PDB 4ZQK). PD-L1 is shown as a light-blue surface, with the CC′FG β-sheet face involved in PD-1 binding highlighted in red. PD-1 is shown as a gray cartoon. This panel defines the functional epitope used as a common structural reference for all subsequent comparisons. (B,C) Antibody-mediated blockade of PD-L1 by therapeutic antibodies. Surface views of PD-L1 bound to durvalumab (PDB 5X8L) and atezolizumab (PDB 5XXY) are shown. Antibody Fabs are displayed as gray cartoons, while the PD-L1 CC′FG β-sheet face occluded by antibody binding is highlighted in red, illustrating direct surface blocking. (D) Macrocyclic peptide inhibition of PD-L1 (pAC65, PDB 8ALX). The peptide is shown as gray sticks binding parallel to the CC′FG β-sheet face of PD-L1. The surface region of PD-L1 contacted by pAC65 is highlighted in red, illustrating antibody-like surface coverage achieved by a compact, preorganized macrocyclic scaffold. (E) Small-molecule–induced PD-L1 homodimerization (BMS-202, PDB 5J89). The two PD-L1 protomers are shown in light blue and gray, respectively. The CC′FG face buried at the dimer interface is highlighted in red, demonstrating indirect neutralization of the PD-1 binding surface through epitope burial rather than direct occlusion. Together, these panels demonstrate that PD-1, therapeutic antibodies, macrocyclic peptides, and small-molecule dimerizers all converge on the same CC′FG β-sheet face of PD-L1. Despite profound differences in molecular modality, inhibition is achieved through a shared geometric solution, differing only in the physical mechanism by which this critical surface is neutralized.

Taken together, the three neutralization modes differ primarily in the degree to which functional inhibition depends on higher-order assembly formation and indirect structural effects. Antibodies achieve direct and extensive surface occlusion of the CC′FG face and currently represent the clinically most validated strategy. Macrocyclic peptides preserve this direct surface-blocking mechanism while introducing conformational preorganization and rigidification within a substantially smaller scaffold. In contrast, biphenyl small molecules neutralize PD-L1 indirectly through ligand-induced homodimerization, requiring productive assembly formation in addition to target engagement. Thus, while all three modalities converge structurally on the same PD-L1 surface, they differ substantially in mechanistic complexity, translational maturity, and developability constraints.

## 7 Design Principles and Outlook

Comparative structural analysis reveals three modality-agnostic design elements: (i) aromatic surface engagement to match the hydrophobic core of the CC′FG face, (ii) peripheral polar or electrostatic anchoring to control orientation, and (iii) explicit management of entropy through preorganization or induced fit. Together, these elements define a unified framework for PD-L1 inhibition (Figure 7). Future progress is unlikely to arise from identifying new allosteric binding sites. Instead, advances will depend on refining how this single surface is engaged, for example by new modalities such as nanobodies, mini proteins or bispecific antibodies. Macrocyclic peptides are particularly well positioned in this regard, as they combine antibody-like geometry with chemical tunability unavailable to biologics and avoid the extreme hydrophobicity often required by small-molecule dimerizers.

**Figure 7:**
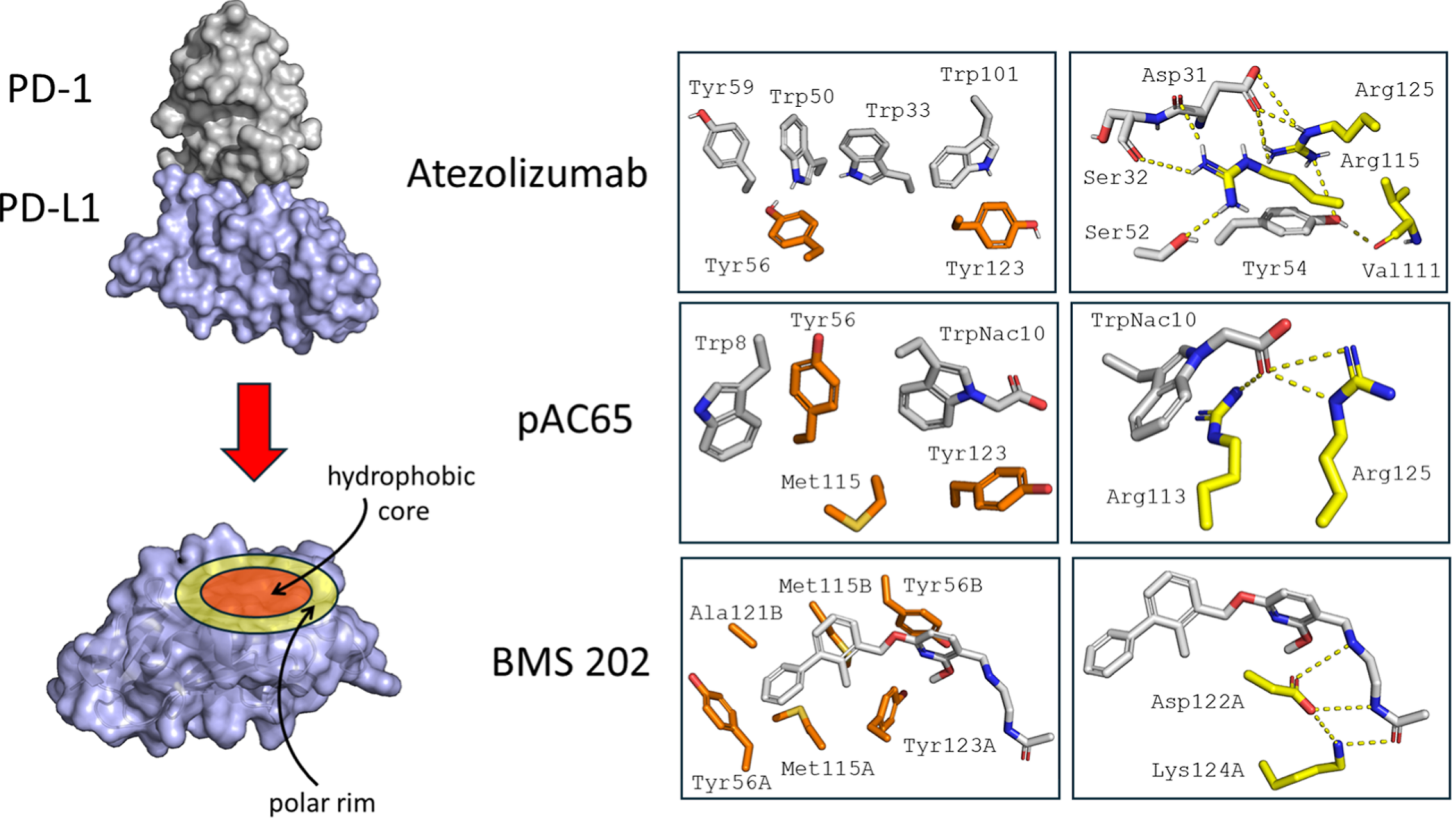
Conceptual summary: three structural modes of PD-L1 neutralization. Schematic representation of the three structural modes by which the CC′FG face of PD-L1 can be neutralized, derived from the comparative analysis of crystal structures shown in Figures 2–7. These modes comprise direct surface blocking by antibodies, surface blocking combined with conformational freezing by macrocyclic peptides, and surface burial through small-molecule–induced PD-L1 dimerization. The schematic distils recurring structural principles observed across PD-L1 complexes. The left column illustrates the hydrophobic core network, highlighting PD-L1-derived residues (orange sticks) and interacting residues from different therapeutic modalities (gray sticks). The right column depicts interactions within the polar rim, emphasizing hydrophilic PD-L1 residues (yellow sticks) and their corresponding modality-derived interaction partners (gray sticks).

These observations suggest several design principles for next-generation PD-L1 inhibitors. Rather than reproducing the entire antibody interface, future modalities will likely benefit from identifying and efficiently recapitulating the minimal interaction hot spots required for productive CC′FG face recognition. Across all modalities, recurrent hydrophobic anchor interactions and strategically positioned polar contacts emerge as the dominant determinants of binding geometry. The challenge therefore shifts from maximizing interface coverage toward compressing the essential recognition elements into smaller, more tunable scaffolds while preserving the spatial organization required for productive PD-L1 engagement. In this context, macrocyclic peptides may represent an important intermediate solution, combining partial antibody-like surface recognition with the conformational preorganization and developability advantages of smaller molecular architectures. Additional emerging modalities—albeit fare from clinical trials—to be mentioned here, including covalent ligands,^23^ degrader-based strategies,^24^ oligonucleotide-derived binders,^25^ and engineered multispecific biologics,^26^ may further expand the therapeutic landscape of PD-L1 modulation. However, in contrast to antibodies, macrocyclic peptides, and biphenyl dimerizers, these approaches currently lack comparable structural and translational maturity across clinically relevant systems. Whether such modalities ultimately converge on the same geometric constraints imposed by the CC′FG β-sheet face remains an important question for future investigation.

## 8 Form Follows Function: Explaining Clinical Asymmetry

Although clinically effective, PD-1/PD-L1 antibodies are associated with immune-related adverse events, underscoring the ongoing need for modalities with potentially different biodistribution and toxicity profiles.^27^ A notable asymmetry emerges when structural mechanisms are considered alongside clinical outcomes. Antibodies targeting PD-1 or PD-L1 are clinically successful, and peptide-based approaches have demonstrated strong translational promise.^28,29^ In contrast, small-molecule PD-L1 dimerizers, despite compelling structural rationale, have not yet achieved comparable clinical validation.^30–33^ This disparity may, at least in part, reflect the principle that “form follows function”. Direct surface occlusion aligns naturally with the biological requirements of PD-L1 inhibition and has proven robust in translation. Antibodies and macrocyclic peptides neutralize the CC′FG face through direct surface engagement, either by steric blockade alone or by combined blockade and conformational rigidification. By contrast, small-molecule biphenyl derivatives rely on an indirect mechanism in which ligand-induced PD-L1 homodimerization buries the PD-1 interaction surface. This mechanism is strongly supported by crystallographic and solution biophysical studies using soluble extracellular-domain PD-L1.20 However, translation of this assembly mechanism into the native membrane context likely introduces additional functional dependencies, including receptor density, membrane topology, trafficking dynamics, and sustained formation of productive higher-order complexes. Thus, the limited clinical translation of PD-L1 dimerizers should not be attributed solely to induced dimerization itself. Compound-specific limitations, including physicochemical properties, pharmacokinetics, dosing, and potential off-target effects, likely also contribute substantially. Nevertheless, compared with antibody therapeutics, these compounds generally exhibit lower target affinities and narrower therapeutic windows, while their reliance on lipophilicity-driven aromatic interactions may increase susceptibility to off-target binding. Together, these observations suggest that indirect neutralization through induced PD-L1 assembly may represent a less translationally robust solution than direct surface engagement of the biologically active CC′FG face.

## 9 Conclusions

Immune checkpoint blockade targeting the PD-1/PD-L1 axis has rapidly become a cornerstone of cancer therapy, with multiple monoclonal antibodies—including PD-1 inhibitors (e.g., pembrolizumab, nivolumab, cemiplimab, dostarlimab, retifanlimab, toripalimab, tislelizumab, penpulimab) and PD-L1 inhibitors (e.g., atezolizumab, avelumab, durvalumab, cosibelimab)—approved across diverse tumor types and indications, reflecting their profound clinical impact in oncology.^34^ Peptides and small molecules targeting PD-1/PD-L1 lag behind antibodies but are intensively investigated in clinical trials. Notably, the clinical progression of the macrocyclic peptide BMS-986189 provides translational validation that antibody-like recognition of the PD-L1 CC′FG face can be achieved within a compact synthetic scaffold. PD-L1 inhibition illustrates how a seemingly intractable flat and large protein–protein interaction can be addressed by diverse molecular modalities that nonetheless converge on a single structural solution. Recognizing this convergence transforms modality diversity into coherent design logic and provides a blueprint for targeting other flat immune checkpoint PPIs, where success will similarly depend on geometric fidelity to biological function rather than on molecular class alone. Emerging modalities such as nanobodies, mini-proteins, engineered multispecific biologics, and alternative scaffold architectures may further diversify the strategies available for PD-L1 neutralization. However, whether these systems ultimately converge on the same geometric constraints and CC′FG-centered recognition principles described here remains an important question for future structural investigation.

## Abbreviations

Å: Angstroms
CD4: cluster of differentiation 4
CDRs: complementarity-determining regions
IgV: immunoglobulin variable domain
MDCII: major histocompatibility complex class II
nM: nanomolar concentration
PD-1: programmed cell death protein-1
PDB: Protein Data Bank
PD-L1: programmed cell death ligand-1
PPIs: protein–protein interactions
TCR: T-cell receptor
